# Patient-reported outcome measures used to improve youth mental health services: a systematic review

**DOI:** 10.1186/s41687-023-00556-0

**Published:** 2023-02-14

**Authors:** Kalpana Thapa Bajgain, Mungunzul Amarbayan, Krystle Wittevrongel, Erin McCabe, Syeda Farwa Naqvi, Karen Tang, Fariba Aghajafari, Jennifer D. Zwicker, Maria Santana

**Affiliations:** 1 Department of Community Health Sciences, Cumming School of Medicine, University of Calgary, Calgary, AB Canada; 2 School of Public Policy, University of Calgary, Calgary, AB Canada; 3 Department of Paediatrics, Cumming School of Medicine, University of Calgary, Calgary, AB Canada; 4 Faculty of Kinesiology, University of Calgary, Calgary, AB Canada; 5 Department of Medicine and Community Health Sciences, University of Calgary, Calgary, AB Canada; 6 Alberta Children’s Hospital, 2888 Shaganappi Trail NW, Calgary, Canada

**Keywords:** Patient-reported outcome measures (PROMs), Child and youth, Mental health conditions

## Abstract

**Background:**

Patient-reported outcome measures (PROMs) are standardized and validated self-administered questionnaires that assess whether healthcare interventions and practices improve patients’ health and quality of life. PROMs are commonly implemented in children and youth mental health services, as they increasingly emphasize patient-centered care. The objective of this study was to identify and describe the PROMs that are currently in use with children and youth living with mental health conditions (MHCs).

**Methods:**

Three databases (MEDLINE, EMBASE, and PsycINFO) were systematically searched that used PROMs with children and youth < 18 years of age living with at least one diagnosed MHC. All methods were noted according to Preferred Reporting Items for Systematic reviews and Meta-Analysis. Four independent reviewers extracted data, which included study characteristics (country, year), setting, the type of MHC under investigation, how the PROMs were used, type of respondent, number of items, domain descriptors, and the psychometric properties.

**Results:**

Of the 5004 articles returned by the electronic search, 34 full-texts were included in this review. This review identified both generic and disease-specific PROMs, and of the 28 measures identified, 13 were generic, two were generic preference-based, and 13 were disease-specific.

**Conclusion:**

This review shows there is a diverse array of PROMs used in children and youth living with MHCs. Integrating PROMs into the routine clinical care of youth living with MHCs could improve the mental health of youth. Further research on how relevant these PROMs are children and youth with mental health conditions will help establish more uniformity in the use of PROMs for this population.

**Supplementary Information:**

The online version contains supplementary material available at 10.1186/s41687-023-00556-0.

## Introduction

Mental health conditions change a person’s thinking, feeling, and behavior, causing discomfort and making it difficult for them to function, comprising 10% of children and youth who experience mental disorders globally [1]. Mental health conditions are the most common cause affecting people’s quality of life in which most mental problems begin before the age of 14 and are frequently misdiagnosed and undertreated; most do not seek help [2]. According to the Mental Health Commission of Canada, “Healthy emotional and social development in our early years lay the foundation for mental health and resilience throughout the lifespan [3].” Youth with mental health illnesses are frequently encountered in various contexts, such as their own families, homes, school, and communities [4]. Often serious problems we come across in adulthood such as depression, substance misuse, family violence, and criminality have roots that start much earlier in life, typically with serious childhood behavior and emotional disorders [5]. Indeed, early detection and intervention can reduce emotional and behavioral problems, and functional impairments, prevent engagement with law enforcement in all forms and improve learning outcomes and school performance [6].

Mental healthcare systems continue developing strategies to improve children and youth's well-being [7]. In patient-centered care, the effectiveness of mental health interventions and improvements can be determined using Patient-Reported Outcome Measures (PROMs) which are carefully assessed and monitored [8]. PROMs are standardized and validated self-administered questionnaires that assess patients’ health and quality of life involving symptoms, function, pain, and physical and mental health [7]. These outcome measures need to be valid, reliable, change-sensitive, important, and meaningful for both the patient and provider [9]. Evidence has shown that the integration of PROMs in routine clinical care practice enriches communication between the patient, family, and healthcare providers, resulting in better care management, maintaining low health service utilization, and patient care experiences and outcomes, and ensuring that the voices of the service user are heard [10, 11]. A study has shown that using PROMs improves treatment outcomes and particularly in Child mental health research, using PROMs in combination with patient feedback allows for faster patient improvement than using PROMs [12, 13]. Despite the evidence that the use of PROMs in clinical care improves health outcomes, their use in mental health settings for children and youth is infrequent in Canada.

Generally, PROMs can be classified into various categories. Generic PROMs are multidimensional and assess the general health aspects that are relevant to the patient group and the general population, allowing comparison across different health conditions, populations, and interventions [14]. Strength and Difficulties Questionnaire (SDQ), and Short Form-36 (SF-36) are some examples of such measures. While disease-specific PROMs aim to gather pieces of information on an aspect of health that is particular for a specific disease [8]. Generic PROMs could be further classified as Profile and Preference-based in which Profile-based measures (for e.g. SF-36) assess the health domains that are measured by multiple items, whereas preference-based measures (such as EQ-5D) determine health conditions and also be used to generate health utility value to calculate quality-adjusted life years (QALYs) which are used in health economic evaluations [15].

The use of PROMs in pediatric care requires specific considerations. For instance, according to the Food and Drug Administration (FDA), while using pediatric PROMs in health research and clinical care special consideration should be granted to the reading level of the child, considering vocabulary, age, and cognition level so that a child can provide a valid and reliable answer [16]. Furthermore, to address some of the age-specific and cognition level aspects, both self-reported and parent-proxy reported PROMs have been employed in pediatric populations [16–20].

A number of PROMS have been developed for the adult and youth population with mental health conditions. There is a consortium of different measures used on different age groups for youth, which implies that there is no consensus in the literature on which measurements are best-practice and most appropriate for this population [21]. Moreover, it is unclear what measures are relevant for use in a clinical context [14]. This study addresses the need to identify measures used across mental health settings and summarizes key properties of the measures relevant for clinical use [22].

The primary aim of this review was to identify appropriate PROMs for children and youth living with mental health conditions (MHCs). The secondary aims were to explore:

1. How PROMs are being currently used in child and youth MHCs such as study design, setting, and in what populations, modes of administration (paper, interview, survey, electronic, and responder type).
2. The psychometric properties (validity, reliability) of the identified PROMs as reported in the studies.

This inventory of PROMs will highlight crucial information for ensuring healthcare is relevant and valuable to children and youth living with MHCs and their families.

## Method

This systematic review is reported following the Preferred Reporting Items for Systematic reviews and Meta-Analysis (PRISMA) guidelines [23]. The search was developed in collaboration with a health librarian and the research team.

### Data sources and selection process

A search of three electronic databases (MEDLINE, EMBASE, and PsycINFO) was performed for articles published from January 2000 to May 2021. Searches were limited from the year 2000 because the integration of PROMs in routine clinical care was initiated after the year 2000 [24].

Search strategies were developed in consultation with a medical health librarian. The first search concept was PROMs, using the following keywords: “Patient-reported outcome measures” OR, “routine outcome assessment” or “self-reported outcome” or “patient outcome assessment” or health-related quality of life” or “PRO” or “PROMs” or “PROMIS”. The second search concept was mental health, searched using the keywords: “Mental health”, “mental illness”, “mental disorder”, OR “mood disorder” or “Schizophrenia” or “eating disorder” or “psychological disorder” or “OCD” or “Depression” or “bipolar or anxiety or “PTSD” or “self-harm”. The third search concept, youth [18 years and younger] was searched using keywords: “Adolescent” or “Youth” or “Teenage” or “Teen” or “children” or “infant” or “kids” or “child” or “toddler” or “juvenile” or “parents” or “caregiver” were used. The three search concepts were combined using the Boolean operator ‘AND’. We also used subject headings in each search concept (MEDline search strategy in Additional file 1: S1). Searches were adapted to each electronic database and limited to the English language.

### Inclusion and exclusion

An article was eligible for inclusion if it: (1) used one or more PROMs (we included PROMs as measurement tools that are validated for use in different settings); (2) was conducted in a population < 18 years of age with at least one MHC with a formal diagnosis according to the DSM 5 [25]; (3) was peer-reviewed; and (4) was published in English and the full text was available. Measures could be completed by children, parents, or both.

Exclusion criteria included: (1) the study did not use a PROM as an outcome measure (including studies evaluating psychometric properties or cultural adaptation of PROMs); (2) the study population did not have a formal MHC diagnosis; (3) study participants were > 18 years or above, (4) the study population had condition related to Neurodevelopmental disabilities (5) also participants diagnosed with medical illness comorbidities i.e. cancer, diabetes, etc. and (6) the full text of the study was not available. Citations generated by all database searches were compiled using Covidence for reference management and data extraction.

During the search, systematic review articles were not included in the final list but were used for a supplementary search. In this supplementary search, citations were extracted, and reference lists were manually examined to confirm the inclusion of all relevant studies. The same inclusion criteria were applied in the supplementary search. The final disagreements about study eligibility were resolved through discussion by the research team.

We only considered PROMs as measurement instruments that are validated for use across different settings (i.e., questionnaires developed and used by a single study were not included). We also included proxy-report PROMs, because in pediatric care, PROMs can also be reported by the family or caregiver of the patient. PROM ‘families’ (i.e., PROMs with multiple forms) were included as well. For example, the Health Utilities Index (HUI) descriptive system can be scored using value sets that provide an HUI‐2 or HUI‐3 index.

### Study screening and selection

To diminish the chances of barring relevant articles and to alleviate bias, four team members worked in pairs (KTB, MMA, KW, and FN) to independently screen titles and abstracts of all studies against our predetermined inclusion and exclusion criteria. The studies which did not meet the requirement for inclusion were eliminated. Any divergences that arose were resolved by senior authors (MS and JZ).

### Data extraction and quality assessment

The following data were extracted from each study that met the inclusion criteria: study characteristics (country and year of publication), study setting, study design, mental health condition, PROM(s) used and type (generic/disease-specific, preference-based), respondent type (self, parent/proxy, or both), response options, number of items, number of domains, domain descriptor, purpose/use of implementation, and the PROM’s psychometric properties (validity, reliability). Four independent reviewers extracted all pertinent data from the articles deemed for inclusion using a standardized form (KTB, MMA, KW, and FN). Methodological quality assessment of the included studies was assessed using the Quality Assessment Tool for the studies with diverse design (QATSDD) critical appraisal tool by the first author (KTB) [26]. The QATSDD demonstrates strong validity and reliability for assessing the quality of quantitative and qualitative studies and for reviewers it may be a useful tool to standardize and increase the rigor of the assessment in their review [26]. Each item in the QATSDD tool is scored on a 0–3 scale (0 = not at all described 1 = very slightly described 2 = moderately described 3 = completely described) with the total score ranging from 0 to 42. The studies with the score above 60% are at low risk of bias whereas studies below 60% are at higher risk of bias. The items comprised a description of an explicit theoretical framework, a statement of objectives in the body of the paper, a detailing of the research setting, a consideration of sample size in analysis, a representative sample, a description of data collection and recruitment procedure, reliability, and validity of measurement of data collection, fit between research question and data collection and analysis methodology, justification for analysis, user involvement in design and strengths and limitation described [26]. For ease of interpretation, the scores were converted into a percentage.

## Results

### Search results

**Fig. 1:**
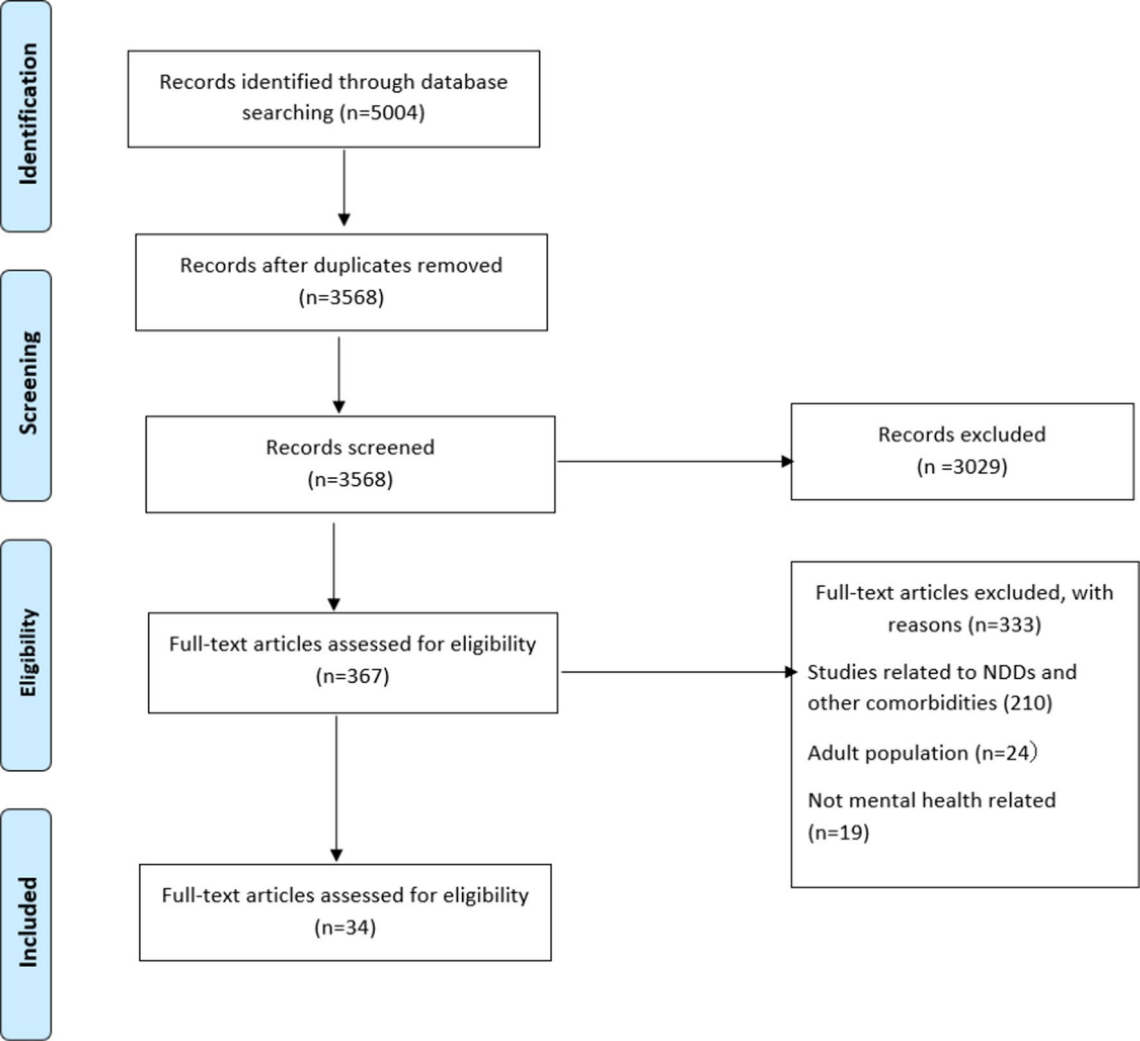
PRISMA 2009 flow diagram

Figure 1 depicts the search result following the PRISMA guidelines [23]. This electronic search returned 5004 articles. After duplicate articles were removed, 3568 articles remained. After title and abstract screening of 3568 articles, 367 potentially relevant studies were identified for full-text screening. Of these, 333 articles were excluded. The reasons for exclusion included: studies related to neurodevelopmental disorders (NDD) and other comorbidities; the age range of participants; and non-mental health-related studies. As a result, 34 articles were included in this systematic review.

### Characteristics of included studies

Overall, we found that in recent years there is growing utilization of PROMs in child and youth mental health settings. Of the 34 studies, 18 studies were conducted in Europe (Germany, Netherland, the United Kingdom, Norway, Austria, Scotland, and Switzerland), while six studies occurred in the United States. Five multi-site studies included more than one country in their evaluation (Table 1).

**Table 1:** General characteristics of included studies

References	Country of origin	Setting	Design	MHC
[27]	Germany	Pediatric Outpatient clinic	Randomized controlled trial	Post-traumatic stress disorder
[28]	Netherlands	Outpatient clinic	Randomized controlled trial	Anxiety
[29]	Canada	Mental health clinics in the pediatric hospital	Observational	General mental disorder
[30]	United States	Pediatric department	Observational	Paraphilia
[31]	Israel	Psychiatric outpatient department	Non-randomized	Major depression
[32]	United Kingdom	Hospital	Observational	Eating disorder
[33]	Austria	Psychiatric outpatient department	Observational	General mental disorders
[34]	Australia	Face to face interview	Observational	General mental disorders
[35]	United States	Outpatient sites	Randomized controlled trial	Bipolar Mania; Schizophrenia; or schizoaffective disorder
[36]	United States	Hospital	Observational	Bipolar 1 disorder
[21]	England, Scotland	Clinic	Observational	General mental disorders
[37]	N/A	Inpatient vs outpatient	Observational	Bipolar 1 disorder
[38]	United Kingdom	Hospital	Randomized controlled trial	General mental disorder
[39]	United Kingdom	Community and clinical setting	Randomized controlled trial	Depression
[40]	United Kingdom	Psychiatric inpatient care	Randomized controlled trial	General mental disorders
[41]	Netherland	Diagnostic center in the hospital	Observational	Avoidant restrictive food intake disorder
[42]	Australia	Clinic	Randomized controlled trial	Anxiety
[43]	Germany	Outpatient psychotherapy treatment	Observational	General mental disorders
[44]	Germany	Outpatient psychotherapy treatment	Observational	General mental disorders
[45]	Norway	Multicentre	Observational	Obsessive–compulsive disorder
[46]	Norway	Multicentre	Observational	Obsessive–compulsive disorder
[47]	USA, Mexico, Russia	Multicentre	Randomized controlled trial	Bipolar 1 disorder
[48]	Chile	PHC	Randomized controlled trial	Depression
[49]	United Kingdom	Child and Adolescent Mental Health Services	Observational	General mental disorders
[50]	United States	Outpatient treatment at multiple sites	Observational	Eating disorder
[51]	Switzerland	National survey	Non-randomized experimental	General mental disorders
[52]	United Kingdom	Multisite	Randomized controlled trial	Depression
[53]	Netherland	Outpatient psychiatric clinic	Observational	General mental disorders
[54]	United States	Multisite (Pediatric clinics)	Randomized controlled trial	Major depressive disorder
[55]	USA, India, Canada, Costa Rica, Mexico	Multisite (hospital, general practice, and academic centers)	Randomized controlled trial	Major depressive disorder
[56]	USA, India, Canada, Costa Rica, Mexico	Multisite (Pediatric clinics)	Non-randomized experimental	Major depressive disorder
[57]	Brazil	Child and Adolescent Psychiatry	Non-randomized experimental	Social anxiety disorder
[58]	United States	Community mental health center or an academic medical center	Observational	Bipolar 1 disorder
[59]	Scandinavian Countries	Clinic	Randomized controlled trial	OCD

Study settings included pediatric outpatient departments and hospitals, mental health clinics, community mental health services, diagnostic centers in the hospital, outpatient psychotherapy treatment centers, and primary health centers. Some studies included multiple sites (pediatric hospitals, general practice, and academic centers). Seventeen studies were observational, 13 were randomized controlled trials, and four were non-randomized experimental studies. In this review 21% of the studies had a diagnosis of major depression, followed by bipolar disorder (12%), anxiety (9%) and obsessive–compulsive disorder (OCD) (9%). Thirty two percent (32%) of the study had unspecified or broad categories of diagnosed MHC, and another 26% were categorised as “Schizophrenia; schizoaffective disorder, eating disorder, avoidant restrictive food intake disorder, paraphilia”.

### Patient-reported outcome measures (PROMs) characteristics

Among 34 studies, there were 28 numbers of unique PROMs identified. Thirteen measures were unique generic profile PROMs, among the most common were KIDSCREEN [29, 43, 44, 48, 51], Strengths and Difficulties Questionnaire (SDQ) [21, 38, 40, 42, 49], KINDL-R [45–47], [58, 59], Pediatric Quality of Life Enjoyment and Satisfaction (PQ-LES-Q) [30, 54–56], and the Pediatric Quality of Life Inventory (PedsQl) 4.0 [33, 34, 41]. There were two generic preference-based PROMs, the Euroqol (EQ-5D 3L and 5L), Health Utilities Index (HUI2/3) and thirteen measures were disease-specific, most of which were used in depression such as beck depression inventory (BDI) and child depression inventory (CDI) (Additional file 2: S2).

### Study population age ranges

The age of the study population ranges from 0 to 18. There are different versions of PROMs existing for different age ranges. Out of 28 PROMs, seven (PQ-LES, CHQ, Pedsqol, CBCL, KIDSCREENN, KINDL-R) were used in children under 8 years. However, the PROMs such as EQ-5D-5L, PROMIS Depression scale, BDI, C-SSRS, SCARED, YMRS, HONOSCA, WEMWBS, CHQ, SDQ, YSR, KINDL-R, CDI have been used across children 12–18 years of age.

### Responder type and method of administration

Many of the PROMs in the included studies were administered to the child for self-report [27–33, 35, 38, 40, 44, 48–50, 52, 54–58]. Twelve measures included both self-report and proxy report [21, 28, 29, 41–47, 51, 58] and five measures have been reported by proxy only [34, 36, 37, 41, 44]. Proxy reporting was used in some studies due to a child’s young age and cognitive abilities (Additional file 2: S2).

Many studies [31, 33–35, 45, 48, 49, 51–53] used non-electronic methods of administration, for example interview, paper in clinic or by mail. Less commonly [38, 41] data were collected using electronic methods whereas most of the studies did not report on methods or modes of PROMs administration.

### Response option/scale

Most PROMs used a Likert scale (three to five points), a linear scale with numbers or words explaining the range or severity of options, yes/no scales, and/or a visual analog scale (VAS). For instance, the KIDSCREEN 27—child version applies a five-point Likert scale from never to always. The PedsQOL 4.0 young child report version (children under the age of 8) utilizes a simplified Likert scale with facial expression response options. The EuroQol is a five-dimensional youth questionnaire (each statement is evaluated from three ordinal levels, no problem to severe problem) and VAS (ranging from best imaginable health to worst imaginable health).

### Number of items

The number of items present on each PROM ranged from five [27, 39, 52] to 118 [43, 44]. Standard descriptive sociodemographic items such as age, sex, date of birth, body functioning, living conditions, and family composition were also included as items in some of the questionnaires (for e.g. Kindle, Kidscreen, TAPQOL), which added to the number of items but did not contribute to the scoring.

### Domain descriptor

Table 2 displays the domains of generic, preference-based, and disease-specific PROMs, the number of PROMs that measure each domain, and the PROM that measures the displayed domain. The most common domains measured were emotional status (mood, emotion, and temper), bodily pain, symptoms and discomfort, social functioning/behaviour (social life, getting along with others, social support, communication, relationship, role function), and physical activities (physical functioning, physical activities, physical wellbeing, and belonging).

**Table 2:** Multidimensional generic, preference-based, and disease-specific PROMs in child and youth mental health concern

Domain assessed									
PROMs	Physical	Pain/discomfort	Daily activities	Emotional status	Social functioning	Negative feeling	Family relationship	Cognitive	Others
*Generic*									
CHQ	✓	✓	✓		✓				
KIDSCREEN	✓		✓		✓		✓		✓
KINDL	✓		✓				✓		
PQ-LES-Q	✓		✓		✓				
PedsQl 4.0	✓		✓		✓		✓		
TAPQOL	✓	✓	✓		✓	✓		✓	
SF-36	✓	✓		✓	✓				
CBCL									
MDBF				✓					
SDQ				✓	✓				
VSP-A									
WEMWBS									
Y-QOL									
YSR				✓					
*Preference based*									
EQ-5D-5L		✓	✓			✓			
EQ-5D-Y		✓	✓			✓			
HUI		✓	✓	✓				✓	
*Disease-specific*									
BDI		✓	✓			✓		✓	
EDE-Q									
CDI		✓				✓			
SCARED		✓				✓			
MFQ				✓					
YMRS		✓							
PROMIS pediatric depression scale	✓		✓		✓				
YSR									
HONOSCA		✓			✓				
SCAS						✓			

Generic PROMs were more focused on the measurement of a broad aspect of health-related quality of life, whereas disease-specific measurements were more specific to psychological assessment and clinical symptoms. For example, generic instruments such as CHQ, KIDSCREEN, KINDRL, PQ-LES-Q, PedsQl 4.0, TAPQOL, CHQ-PF-50, and SF-36 also covered the domain of physical activities, daily activities, and social functioning/ behavior while KIDSCREEN, KINDRL, PQ-LES-Q also included family relationships. Preference-based PROMs included pain/discomfort, daily activities, emotional status, and negative feeling. while disease-specific PROMs, including SCARED-R, CDI, BDI-II, YMRS, HONOSCA, assessed bodily pain, discomfort, and symptoms. Among these specific PROMs, SCARED-R, SCARED-D, BDI, SCAS were specifically developed to measure mental health in children and youth including items on negative feelings. Likewise, the other common dimensions covered are family and friend relationships, school and leisure achievement, symptomatology, mental health, change in health, and self-esteem.

### Psychometric properties

Table 3 summarizes the evidence of psychometric properties (validity, reliability) of PROMs that have been reported by the studies in this review. Out of 34 studies, some studies reported on information on more than one psychometric property and included more than one PROMs in their studies. Forty-two percent of the studies (42%) reported on a type of validity, and the internal consistency (Cronbach’s alpha). The rest did not report any information or made a general statement that the measures were valid and reliable. Moreover, thirty five percent (35%) of studies of the identified PROMs were reported to have good test–retest reliability, Intraclass correlation coefficient, or Guttman split-half reliability of PROMs identified while the rest of the studies did not report any information on reliability.

**Table 3:** Psychometric properties of PROMs (validity, reliability, and responsiveness)

PROM name	Validity	Reliability	Responsiveness
EQ-5D-5L [27]	NR (Not Reported)	N/R	N/R
EQ-5D-5L [52]	NR	N/R	N/R
Screen for Child Anxiety Related Disorders [31]	NR	N/R	
Screen for Child Anxiety Related Disorders [28]	Internal consistency (0.92–0.96)	Good test–retest reliability (intraclass correlation coefficients = 0.34–0.79)	N/R
Screen for Child Anxiety Related Disorders Child version [57]	Internal consistency (alpha = 0.74–0.93)	Test–retest reliability (intraclass correlation coefficients = 0.70–0.90)	N/R
Screen for Child Anxiety Related Disorders Parent version [57]	Internal consistency (alpha = 0.74–0.93)	Test–retest reliability (intraclass correlation coefficients = 0.70–0.90)	N/R
The Children's Depression Inventory—2 [52]	Internal consistency (Cronbach’s alpha = 0.91)	Test–retest reliability (0.76–0.92)	N/R
The Children's Depression Inventory—2 [28]	Internal Consistency (0.75–0.85)	N/R	N/R
KIDSCREEN-27 [29]	Internal consistency (0.67–0.88)	N/R	N/R
KIDSCREEN-27 [44]	N/R	N/R	N/R
KIDSCREEN-27 [43]	Internal consistency: (0.78–0.83)	Intraclass correlation between ICC = 0.44–0.61	N/R
KIDSCREEN-27 [48]	The internal consistency (0.80–0.84)	Intraclass correlation between ICC = 0.44–0.61	N/R
KIDSCREEN-27 [50]	Internal consistency: (0.78–0.88)	N/R	N/R
Pediatric Quality of Life Enjoyment and Satisfaction Questionnaire [30]	NR	NR	Kevin’s scores [53–69], correspond with the 68th and 96th percentiles
Pediatric Quality of Life Enjoyment and Satisfaction Questionnaire [54]	NR	NR	NR
Pediatric Quality of Life Enjoyment and Satisfaction Questionnaire [55]	NR	NR	NR
Pediatric Quality of Life Enjoyment and Satisfaction Questionnaire [56]	NR	NR	NR
PROMIS Pediatric Depression Scale [30]	NR	NR	Liebowitz Social Anxiety Scale for Children/Adolescents decreased from (69–43) and (65–30)
Beck Depression Inventory [31]	NR	NR	NR
Beck Depression Inventory-II [32]	NR	NR	Patients had a high level of depression (BDI: 38.1 ± 15.6 vs. 26.6 ± 12.4) and a higher rate of suicidal behavior the beginning. All physical and psychosocial measures improved substantially and clinically significantly by discharge
Beck Depression Inventory-II [39]	N/R	N/R	The depression score reduced after receiving CCBT
Beck Depression Inventory-II [48]	N/R	N/R	N/R
Columbia Suicide Severity Rating Scale [31]	NR	NR	No differences were detected between the group of children who had a previous SA
Suicide Ideation Questionnaire Short Version [31]	NR	NR	NR
Young Mania Rating Scale [31]	NR	NR	NR
The health of the Nation Outcome Scale for Children and Adolescents [32]	NR	NR	Children’s Global Assessment Scale: 13.6 + 2 versus 26.9 + 9;(HONOSCA): 41.7 + 5 versus 31.9 + 5) were worse at the beginning. All physical and psychosocial measures improved substantially and clinically significantly
Morgan-Russell (M-R) scale [32]	NR	NR	NR
Multidimensional Mood Questionnaire [33]	NR	NR	Significant improvement in current mood state, dimension’s mood (mean 1.86; 95% CI 0.13, 3.58; *p* = 0.036),calmness (mean 2.71, 95% CI 1.07, 4.36; *p* = 0.002)
Pediatric Quality of Life Inventory [34]	NR	NR	N/R
Pediatric Quality of Life Inventory [33]	N/R	NR	No changes in quality of life between 2 groups
Pediatric Quality of Life Inventory [41]	Internal consistency (Cronbach alpha: > 0.70)	NR	NR
Warwick-Edinburgh Mental Well-Being Scale [33]	N/R	NR	No changes in well being were found
Child Health Questionnaire [35]	Internal consistency (Cronbach α:0.70–0.87)	NR	NR
Child Health Questionnaire Parent Form [36]	NR	NR	Significant improvement in HRQOL, particularly psychosocial subscale
Child Health Questionnaire Parent Form [37]	N/R	N/R	N/R
Strengths and Difficulties Questionnaire [21]	Internal consistency (α: 0.73)	NR	N/R
Strengths and Difficulties Questionnaire [38]	N/R	NR	Economic analyses suggest that SDS has at least a 50% probability of being cost-effective compared with usual care
Strengths and Difficulties Questionnaire [40]	Internal consistency (α: 0.73)	NR	N/R
Strengths and Difficulties Questionnaire [42]	Internal consistency (α: 0.73)	NR	Significant improvements in child-reported emotional problems from pre-treatment to post-treatment
Strengths and Difficulties Questionnaire [49]	N/R	NR	NR
European Quality of Life Five Dimension [39]	N/R	NR	Quality of life was improved
Health Utility Index Mark 2 [39]	N/R	NR	NR
Mood and Feelings Questionnaire [39]	Internal consistency (α = 0.95)	NR	The reduction was seen in depression score as measured by MFQ
Spence Children’s Anxiety Scale [39]	Internal consistency (α = 0.92)	Guttman split-half reliability (0.90)	NR
Spence Children’s Anxiety Scale [42]	Internal consistency (α = 0.92)	Guttman split-half reliability (0.90)	Significant improvements in child-reported anxiety from pre-treatment–posttreatment
TNO-AZL Preschool Children Quality of Life [41]	Cronbach's alpha (0.71–0.92)	NR	NR
Child Behavior Checklist [44]	NR	NR	NR
Youth Self Report [43]	NR	NR	NR
KINDL-R [46]	Cronbach’s alpha = ≥ 0.70	NR	Age-specific versions take into account the changes in the quality-of-life dimensions throughout the child’s development
KINDL-R [45]	Cronbach’s alpha = ≥ 0.70	NR	NR
KINDL-R [47]	Internal consistency = 0.84–0.89	NR	No change in environmental conditions, while change did significantly impair the quality of life of children
KINDL-R [58]	Internal consistency (Cronbach’s α ≥ 0.70)	NR	NR
KINDL-R [59]	Cronbach’s α = 0.80) parent ratings, (0.82) the child ratings	NR	NR
Eating Disorder Examination-Questionnaire [50]	N/R Cronbach's α: Restraint = 0.82; Eating Concern = 0.81; Shape Concern = 0.92; Weight Concern = 0.83, and Global score = 0.95	NR	NR
36-Item Short Form Survey [50]	Widely used and well-validated	N/R	NR
EQ-VAS [52]	N/R	N/R	NR
Social Phobia and Anxiety Inventory for Children [57]	Internal consistency (α = 0.946)	The test–retest reliability (r = 0.780)	NR
Youth Quality of Life Instrument–Research Version [55]	Internal consistency (α = 0.77–0.96)	Intra-class-correlation coefficient (ICCs = 0.74–0.85)	NR
Children's Yale-Brown Obsessive–Compulsive Scale [59]	Internal consistency (α = 0.90)	Intra-class-correlation coefficient (ICC = 0.79)	Significant differences between the first and second administrations for the Obsession Severity Score with administration being lower than those on the first

*NR* not reported

### Risk of bias assessment

In accordance with the QATSDD tool, the quality rating of included studies ranged from 38 to 90% with an average quality rating of 65%. Twenty-nine studies score above 60% (See QATSDD score in Additional file 3: S3). Overall, the studies possessed a high score on the explicit theoretical framework, statements of aim/objective, clear description of research questions, reasonable sample size, good justification of analytical methods, and strengths and limitations critically discussed. On the other hand, some criteria consistently received lower scores across studies, including a limited description of the research setting, evidence of sample size, and evidence of user involvement in design.

## Discussion

In this systematic review, twenty-eight PROMs were identified from 34 studies to improve child and youth mental health services. We described both generic and disease-specific PROMs used in this population, their psychometric properties, and how they were most commonly used.

This study finds that PROMs used in child and youth mental health settings are more predominant in European countries; showed more than half of the studies were conducted in European countries including the United Kingdom, Germany, Netherland. These findings are consistent with the study which also showed outcome measures were frequently used in child and adolescent mental health services in the United Kingdom, Norway, and Denmark [60]. Another literature review also showed that England, the Netherlands, Sweden, and the United States are the nations that are advanced in implementing PROMs at the national level, with growing interest in adopting a national strategy in Canada [61].

Generic PROMs are multidimensional and assess the general aspects of health that are relevant to the patient group and the general population, allowing comparison across different health conditions, populations, and interventions [14]. The KIDSCREEN, SDQ, KINDL, PedsQL were our review’s most used generic PROMs. Well-validated generic PROMs such as SDQ, SF-36, and CHQ were used in mental health settings and have been reported in other reviews [62]. Typically, generic PROMs lack sensitivity to disease-specific outcomes and tend to be used in the general population and are perhaps more relevant at the system level [8, 63]. On the other hand, generic preference-based measures such as EQ-5D and HUI are the most common preference-based PROMs identified in this review, and the same finding has been revealed in a previous review [64]. These measures are specifically developed for the youth and adolescent population, maybe the cause of their lower frequency of usage in the literature might be due to less vigorous data on their psychometric properties [65].

Disease-specific PROMs are rather focused to assess the aspect of health that is particular for a specific disease [8]. Like generic PROMs, our review revealed various disease-specific measures have been used for this population. This is somewhat unsurprising as research shows that in recent years usage of disease-specific measures has increased at an exponential rate [66]. Disease-specific PROMs are focused on the individual and are therefore most applicable for assessing specific treatment outcomes in defined populations [8, 63]. Ultimately, generic and disease-specific PROMs provide complementary information, so it is recommended to use both to obtain the full information that is required to support health systems [67].

Another important consideration is the “responder type”, which can be either self, parent/caregiver, or both, and from a treatment point of view, all these viewpoints are essential [21]. It is recommended to use the proxy version, although our review disclosed very little use of parent reports including wide age ranges of children and youth. However, using self and proxy-reported versions determines various limitations, perhaps due to differences in thinking levels about the events and interpretation in different ways, though it seems that the proxy measurement is very useful [68, 69]. Many of the studies included in this review used paper-based methods of administration, which is the traditional common way to administer. However, electronic methods of data collection are becoming more popular [70] to reduce the extra burden of data entry, and they have the potential to be more cost-effective in the long run, in comparison to paper-based methods [71].

Concerning the response scale, it should be easily understandable and obvious for children, particularly with mental health concerns. The current review showed that most of the questionnaires used the Likert scale in some form. This finding is consistent with Davis et al. [72], that there is some evidence for children aged 8 and up, that the five-response option is likely to be valid, younger children seem to be differentiated and often go for severe options when compared with the parents. On the other hand, using facial expressions or pictorials for assessing pain or discomfort is common and assumed to be easily readable or understood by children [72]. Some of the studies concluded that pediatric questionnaires were quicker to complete if illustrations were provided and presumed that pictorial could assist children’s interest, capture their attention, clarifying response options which will ultimately create a more meaningful response. Further investigation is recommended, on whether this pictorial aid works better in the real world, however, pictorial might be beneficial for younger children based on expert opinion and many instruments that have adopted this method [73].

In this review, we found the instruments have provided an accepted standard of internal consistency, and fairly a few specified data on reliability, and responsiveness and we did not explicitly include studies that evaluated the psychometric properties of PROMs. For the clinical trial, longitudinal studies, or monitoring patients it’s concerning when there is a lack of evidence in responsiveness [66]. This highlighted whether the instruments can identify clinical change over time or not. Notably, we presented the typical concept of validity, reliability of the measures in the study. Mokkink et al. [74] have encouraged to follow COSMIN guidelines while developing PROMs with appropriate validity and a high degree of evidence [74]. This could be taken into consideration for future review.

There are certain challenges have been recognized in using PROMs in this population. As reported elsewhere, the content and format of PROMs are not able to capture or convey the complexity of the youth’s experience [75, 76]. Clinicians are more concerned about the technical aspect of PROMs use as it could diminish the time for therapy during an encounter [77]. Other commonly argued barriers to PROMs use include constraints around time, allocation of resources and training, and perceived dearth of clinical utility [68].

Overall, we found that in recent years there is growing utilization of PROMs in child and youth mental health settings. We identified inconsistencies across studies regarding the use of measures as several measures used with different age groups implies that there is no consensus on best practice and most appropriate measures targeted for this population [68]. However, disease-specific PROMs have been upsurging, specifically, since the increase in the production of such measures in 2001 [66]. Future studies need to attention on the improvement of the PROMs designed especially for child and youth with MHC.

### Limitations

This study is not without limitations. Given that grey literature was not studied, there is the potential that some PROMs may have been missed. However, our search strategy was otherwise complete and comprehensive given our broad inclusion criteria and use of a research librarian. In addition, we reviewed the reference lists and citations of included studies and hand-searched all identified prior reviews for potentially eligible studies. In addition, despite our search being limited to three electronic databases, this is not likely to have affected the comprehensiveness of our search given that these databases are the most relevant in capturing mental health outcome measures [14, 78]. In our study, the quality of the studies was assessed but conducted independently, without inter-related reliability measures calculated and quality appraisal did not impact the study selection. We also did not include PROMs that were still in the phase of validation, over time they would have met the criteria with more extensive validation.

## Conclusion and future research

This review provides an overview of the PROMs available for children and youth living with MHCs and provides evidence on the type and measurement characteristics of these PROMs.

Moreover, the evidence from this review can be used to inform clinical practice and patient and family-centered care. There is a growing interest in applying PROMs to engage patients in the decision-making process and to help health care professionals to make better decisions about their treatment [8]. This systematic review informs our research program about integrating PROMs into routine clinical care of youth living with MHCs aiming to improve the mental health of youth. Further research is needed to evaluate the plausibility of integrating these measures into routine clinical care and mental health research.

## Supplementary Information

**Additional file 1.** Search strategy for PROMs used in child and youth mental illnesses: Supplementary file (S1): Search strategy (MEDLINE).

**Additional file 2.** General Characteristics of the included studies.

**Additional file 3.** Quality assessment of the included studies.

## Data Availability

All data generated and analysis during this review are included in this published article (and its supplementary information file). The original contributions presented in the study are included in the article, further inquiries can be directed to the corresponding author/s.

## Abbreviations

CHQ: Child Health Questionnaire
KIDSCREEN 25: KIDSCREEN 25
KINDR-L: KINDR-L
PQ-LES-Q: Pediatric Quality of Life Enjoyment and Satisfaction
PedsQ l 4.0: Pediatric Quality of Life Inventory 4.0
TAPQOL: TNO-AZL Preschool Children Quality of Life
CHQ-50: Child Health Questionnaire-Parent Form-50
SF-36: 36 Items Short Survey
PROMIS: Patient-Reported Outcomes Medical Information System
(EQ-5D-5L, EQ-5D-Y): European Quality of Life Five Dimension
HUI Mark2/3: Health Utility Index
SCARED-R: Screen for Child Anxiety Related Disorders
CDI: The Children’s Depression Inventory
BDI-II: Beck Depression Inventory
YMRS: Young Mania Rating Scale
HONOSCA: The Health of the Nation Outcome Scale for Children and Adolescents
EDEQ: Eating Disorder Examination Questionnaire
SDQ: Strength and Difficulties Questionnaire
MDBF: Multidimensionnel Mood Questionnaire
MFQ: Mood and Fatigue Questionnaire
SCAS: Spence Child’s Anxiety Scale
YSR: Youth Self Report
