# Supplementary material for: Patient-reported outcome measures used to improve youth mental health services: a systematic review
Source: J Patient Rep Outcomes. 2023 Feb 14;7:14. doi: 10.1186/s41687-023-00556-0 (PMC9928989; doi:10.1186/s41687-023-00556-0)
Supplement: Supplementary file 1 — Additional file 1. Search strategy for PROMs used in child and youth mental illnesses: Supplementary file (S1): Search strategy (MEDLINE). [file 41687_2023_556_MOESM1_ESM.docx]

**Search strategy for PROMs used in child and youth mental illnesses:**

**Supplementary file (S1): Search strategy (MEDLINE)**

1. exp Patient Reported Outcome Measures/

2. (patient-reported outcome* or self-reported outcome* or patient outcome assessment* or health- related quality of life* or PRO or PORMs).kf,tw.

3. 1 or 2

4. exp Mental Health Services/

5. exp mental health/

6. (mental illness* or mental disorder*).kf,tw.

7. (mood disorder* or Schizophrenia* or eating disorder or psychological disorder* or ADHD* or OCD* or Depression or bipolar or anxiety or PTSD* or self-harm).kf,tw.

8. 4 or 5 or 6 or 7

9. (Adolescent* or Youth* or Teenage* or Teen* or children* or infant* or kids* or child* or toddler* or juvenile* or parents* or caregiver*).kf,tw.

10. 3 and 8 and 9

11. limit 12 to (english language and yr="2000 -Current")
